# 5 mm versus 10 mm umbilical port during laparoscopic cholecystectomy: do outcomes justify broader use in obese patients? A randomized controlled trial

**DOI:** 10.1007/s00464-025-12016-5

**Published:** 2025-08-06

**Authors:** Islam M. Korayem, Amr S. Bessa, Rashed W. Hassan

**Affiliations:** 1 Department of Surgery, HPB and Digestive Surgery Unit, Faculty of Medicine, Alexandria University, Alexandria, Egypt; 2 Faculty of Medicine, Alexandria University, Alexandria, Egypt

**Keywords:** Mini-laparoscopic cholecystectomy, 5-mm umbilical port, Portsite hernia

## Abstract

**Background:**

Mini-laparoscopic cholecystectomy (MLC) is proposed as a modification for standard laparoscopic cholecystectomy to improve patient experience and postoperative outcomes. The aim of this study is to assess the feasibility and outcomes of performing laparoscopic cholecystectomy using 5 mm umbilical port and telescope in obese patients who are at higher risk for developing port-site complications while factoring in surgeon’s satisfaction.

**Methods:**

240 obese patients with chronic calcular cholecystitis were randomly allocated to two groups, standard laparoscopic cholecystectomy (SLC) group using 10 mm umbilical port, and MLC group using 5 mm one. The groups were analyzed for umbilical port-site hernia (PSH), postoperative pain, total operative time, and patient and surgeon satisfaction.

**Results:**

There were no conversions from MLC to SLC. None of the patients in the MLC group experienced port-site hernia compared to 6 patients in the SLC group (*P* = 0.03). MLC group had shorter total operative time (46 vs 52 min, *P* = 0.005), less pain during the first 24 h postoperative (*P* < 0.001), and fewer in-hospital analgesic requirements (*P* < 0.001). Duration of postoperative analgesia at home was shorter in MLC group (*P* < 0.001) with earlier return of patients to normal daily activities compared to SLC group (*P* < 0.001). Patient satisfaction and cosmesis were significantly higher among the MLC group (*P* < 0.001), however, surgeon satisfaction was inferior with the use of 5 mm telescopes compared to 10 mm ones (*P* = 0.06).

**Conclusion:**

Mini-laparoscopic cholecystectomy using 5 mm umbilical port and 5 mm telescope is safe and feasible among obese patients with uncomplicated gallstone disease. It offers shorter operative time, less postoperative pain and analgesic requirement, earlier return to normal daily activities, lower incidence of port-site hernia, and higher degree of patient satisfaction.

**Supplementary Information:**

The online version contains supplementary material available at 10.1007/s00464-025-12016-5.

Introduced in 1989, laparoscopic cholecystectomy (LC) revolutionized the surgical management of symptomatic gallstone disease and became the gold standard. [1–3] Being minimally invasive procedure, it offers less postoperative pain, shorter length of hospital stay, faster recovery with earlier return to daily activities, and superior cosmetic outcomes. [4, 5] Conventional LC employs the use of four ports, two 10 mm and two 5 mm ports. With improved technology and advancing surgical experience, several modifications advocating reduction of the size and/or number of the ports have evolved and became referred to as mini-laparoscopic cholecystectomy. [5–8] Such innovations aimed to further minimize the degree of surgical trauma to improve patient experience in terms of pain and cosmetic outcome. [9]

Port-site hernia (PSH) is a rare, however, known complication following laparoscopic abdominal surgeries with incidence ranging between 0.7 and 6% which could reach up to 22%. [10–12] Well established risk factors for PSH include umbilical port location, ports ≥ 10 mm in size - even with fascia closure, obesity, and wound infection. [13–15] On the other hand, ports < 10 mm in size are rarely associated with PSH and thus do not require routine facial closure. This is explained by their relative smaller size which predispose to limited tissue trauma with better preservation of fascial integrity. [11, 13, 14, 16] 

Data is scarce with regards to performing MLC in obese patients. Accordingly, we sought to investigate the feasibility and outcomes of MLC using reduced 5 mm umbilical port and 5 mm telescope among obese patients while addressing the extent of patient and surgical team satisfaction.

## Materials and methods

### Study design

Between January 2023 and April 2024, all patients ≥ 18 years with symptomatic gallstone disease were screened for inclusion in the study. Exclusion criteria were body mass index (BMI) < 30 kg/m^2^, acute calculous cholecystitis (ACC), gallstone disease complicated by common bile duct (CBD) stones, acute cholangitis or acute pancreatitis at time of presentation, presence of paraumbilical hernia, and history of prior midline or upper abdominal surgery.

All patients with uncomplicated chronic calcular cholecystitis eligible for enrollment were assessed for fitness to undergo laparoscopic cholecystectomy based on the guidelines of the American Society of Anesthesiologists (ASA). They were then randomly allocated into 2 groups based on the size of the umbilical port used: standard laparoscopic cholecystectomy (SLC) group using 10 mm umbilical port and 10 mm telescope, and mini-laparoscopic cholecystectomy (MLC) group using 5 mm umbilical port and 5 mm telescope. Randomization was performed using the sequentially numbered opaque and sealed envelope (SNOSE) technique.

The study was approved by the Ethics Committee and Institutional Review Board of Alexandria University Faculty of Medicine (IRB No. 00012098, FWA No. 00018699) and registered with serial number 0306192. Every participant provided a signed informed consent document after thorough explanation of the procedure and outcomes while addressing all patient concerns in compliance with the Declaration of Helsinki.

### Preoperative assessment

Patients’ demography, associated comorbidities, and history of prior lower abdominal/pelvic surgeries were collected. Preoperative laboratory investigations included total and direct serum bilirubin, alanine aminotransferase (ALT), aspartate aminotransferase (AST), alkaline phosphatase (ALP), gamma-glutamyl transferase (GGT), C-reactive protein (CRP), hemoglobin (Hb) level, platelet count, total leukocyte count (TLC), and polymorphonuclear leukocyte (PMNL) count. Abdominal ultrasound was routinely performed to achieve the diagnosis of chronic calcular cholecystitis with focus on the number and size of gallstones. Magnetic resonance cholangiopancreatography (MRCP) was performed whenever elevated bilirubin or abnormal liver enzymes were encountered to exclude CBD obstruction.

### Operative details

**Fig. 1:**
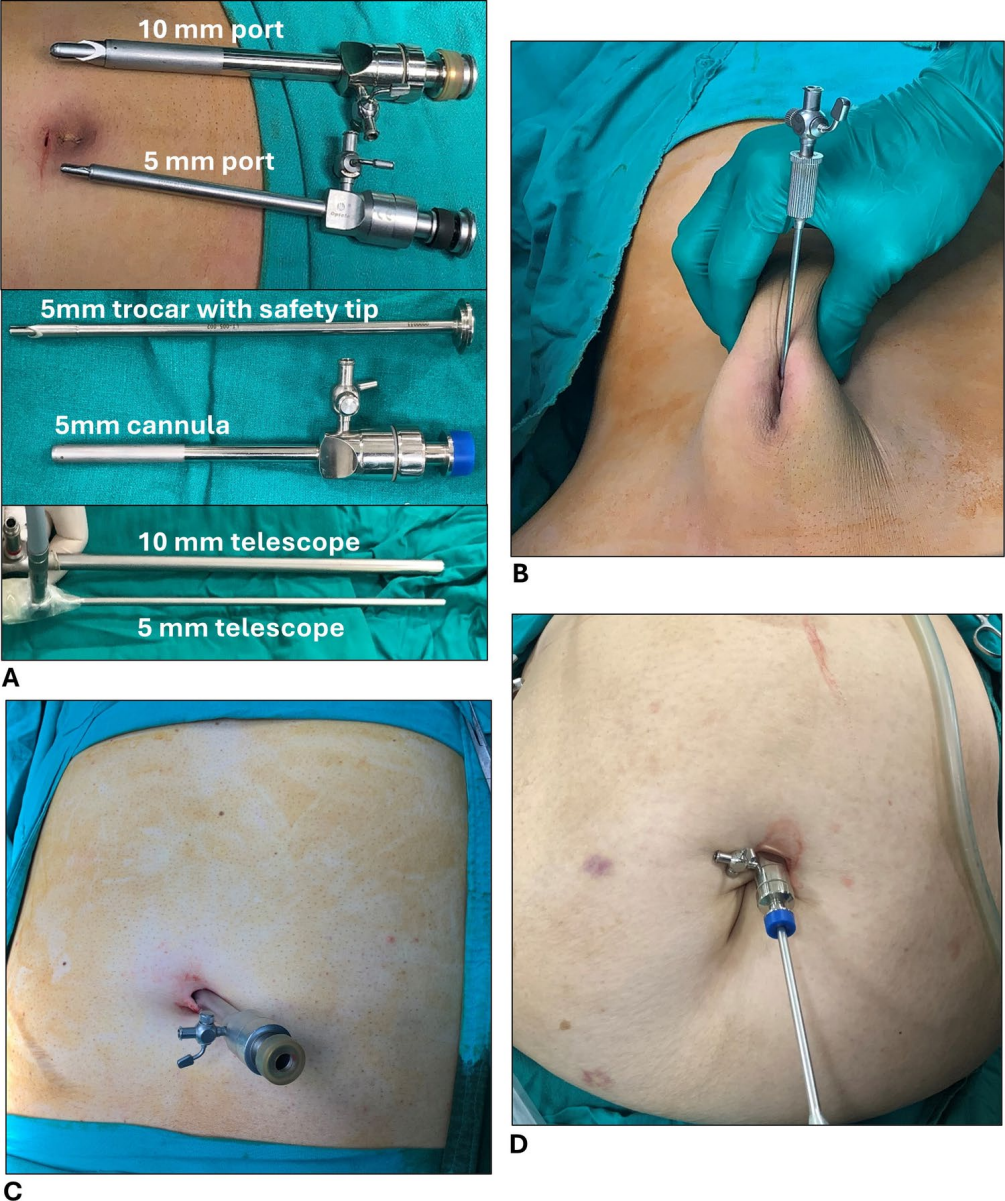
**A** 5mm vs 10mm metal trocar/cannula with safety tip and corresponding telescopes, **B** Veress needle inserted through supraumbilical wound to achieve closed pneumoperitoneum, **C** 10 mm umbilical port blindly inserted, **D** 5mm umbilical port after blind insertion with 5mm telescope

All surgeries were performed under general anesthesia by the same team of qualified and experienced surgeons in the field of laparoscopy and hepatobiliary surgery to maintain standardization of the technique across all patients. Prophylactic 1 g Ceftriaxone was given intravenously within 30 min prior to skin incision. Patients were placed in a reverse Trendelenburg position with right shoulder rotated upward by 30°. Autoclavable metal ports and trocars with safety spring recoil tip (Fig. 1A) were routinely used to ensure safe trocar insertion.

**Fig. 2:**
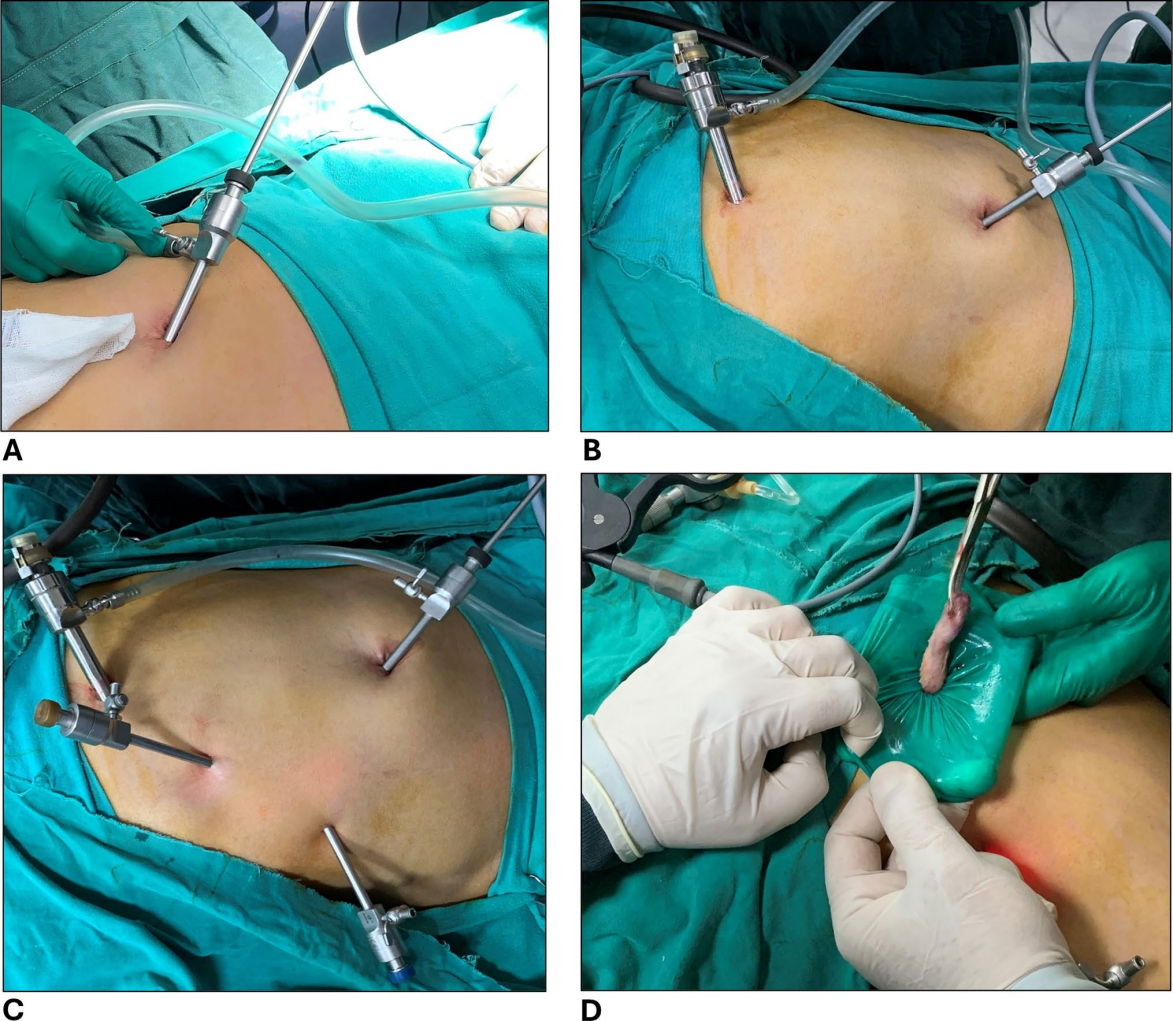
**A** Gas hose connected initially to 5mm umbilical port, **B** gas hose switched to 10mm epigastric port after insertion under direct vision, **C** the final 5–10–5–5– port-pattern in MLC group, **D** GB retrieval in custom-made specimen bag through epigastric port-wound

Closed pneumoperitoneum was created using Veress needle applied through tiny supraumbilical incision as shown in Fig. 1B. Once abdominal pressure reached 10–12 mmHg, the needle was removed and blind insertion of the umbilical port followed while lifting the abdominal wall upward to guard against visceral or vascular injury. In SLC group, 10 mm umbilical port was used (Fig. 1C) with standard 10 mm telescope, whereas a 5 mm port and 5 mm telescope were used in MLC group (Fig. 1D). The remaining 3 ports were similar among both groups and introduced under direct vision in the following order: 10 mm epigastric port slightly to the right of midline, 5 mm subcostal port at the midclavicular line, and another 5 mm port at the anterior axillary line. In the SLC group, the gas tubing was kept connected to the 10 mm umbilical port throughout the entire surgery. However, in the MLC group, it was initially connected to the 5 mm umbilical port as shown in Fig. 2A to maintain pneumoperitoneum until the 10 mm epigastric port was inserted to which it was then switched as shown in Fig. 2B to allow for more rapid and efficient insufflation. The final pattern of the ports in SLC and MLC groups was 10–10–5–5 and 5–10–5–5, respectively (Fig. 2C).

Gallbladder (GB) dissection and extraction maneuver were similar in both groups and followed our standardized technique. [17] Briefly, the hepatocystic triangle of Calot was cleared to achieve the critical view of safety to ensure proper identification and control of cystic duct and cystic artery. GB was then dissected free from the liver bed, placed in a custom-made specimen bag, and retrieved through the epigastric port (Fig. 2D). Abdominal drain insertion was not routine in our practice unless extensive adhesiolysis, subtotal cholecystectomy, or conversion to open surgery were performed. Care was taken to properly evacuate the abdominal cavity from insufflated carbon dioxide gas, especially in the MLC group owing to the smaller umbilical port which was the last port remaining after removal of other ports under vision. All of the 5 mm ports, including the 5 mm umbilical port in the MLC group, required closure of skin only using 3/0 absorbable suture material (Supplemental Fig. 1A and 1B). Fascial closure was performed for the 10 mm umbilical ports in the SLC group using 2/0 absorbable suture material through laparoscopic fascial closure instrument under vision followed by subcutaneous tissue and skin closure.

Any intraoperative events such as bleeding or visceral injury were recorded. The need for conversion from MLC to conventional SLC was recorded with statement of the reason. The total operative time was measured from the time of first skin incision till closure of the last port-site wound. For accurate analysis, we divided it into 2 intervals:

- Time required for abdominal access, GB dissection, and extraction which spans from first skin incision to complete retrieval of the GB.
- Time required for closure of the 4 port-site wounds after GB retrieval.

### Postoperative pain management and follow up

All patients were placed on standard and flexible protocol for postoperative pain control during the first 24 h postoperative consisting of 12-hourly intravenous administration of 30 mg ketorolac which could be increased to 8-hourly dose upon patient request. Should further analgesia be required beside ketorolac, an intravenous bolus dose of 1 g paracetamol was provided.

Postoperative pain was assessed and recorded by the same surgeon at 2-, 6-, 12-, and 24-h intervals during the first 24 h postoperative using visual analogue scale (VAS) ranging from 0 to 10 with 0 for no pain and 10 for the worst imaginable pain perceived.

All patients were planned for discharge 24 h after surgery provided that they tolerated pain and oral intake. First follow-up visit was scheduled on postoperative day 4 for physical assessment and wound dressing (Supplemental Fig. 1C and D). Port-site specific pain score was also assessed during the first visit. Second follow-up visit was scheduled on postoperative day 7. The duration of postoperative analgesic requirement at home was recorded as well as the time required by patients to return to their normal daily activities/work. A minimum follow-up period of 1 year was planned for all patients to assess for the incidence of PSH. Accordingly, subsequent follow-up visits were scheduled at 1-, 3-, 6-, and 12-months postoperative. When suspected, PSH was initially assessed by clinical examination and focused abdominal wall ultrasonography. If the hernia was difficult to appreciate clinically and/or the ultrasound report was inconclusive, computed abdominal tomography was performed. During the whole follow-up period, unplanned visits were resorted to whenever surgical complications requiring further care were encountered. Postoperative complications were recorded and reported using the Clavien–Dindo classification. [18, 19]

### Patient and surgical team satisfaction

A 5-point Likert scale survey model [20] was used to gather patient and surgical team satisfaction. Patient satisfaction focused on umbilical port wound size, associated pain, wound complications, and cosmetic outcome. They were recorded at 1-, 3-month, and 6-month postoperative and the mean reading was taken. Surgical team satisfaction addressed the team’s experience with the use of 5 mm vs 10 mm umbilical port and telescope with regards to the visual field, image quality in terms of brightness, resolution, and accuracy of fine anatomic details. The ease of navigating the optical system by the assistant surgeon in obese patients was also assessed.

### Study endpoints

Umbilical PSH was our primary endpoint. Secondary endpoints included operative time, postoperative pain, 24-h analgesic requirement, duration of analgesic intake at home, time required to return to normal daily activity, and patient and surgical team satisfaction.

### Statistical analysis

Normality of data was assessed using Shapiro–Wilk and Kolmogorov–Smirnov tests. Descriptive statistics were displayed as numbers and percentages for categorical variables and as medians with interquartile ranges (IQR) for continuous variables. Group comparisons were conducted using Pearson’s Chi-square/Fisher’s exact test for categorical variables and Mann–Whitney U test for continuous variables with statistical significance set at *P* < 0.05. All statistical analyses were conducted using IBM® SPSS® Statistics version 26 (IBM Corporation, Armonk, NY).

## Results

**Fig. 3:**
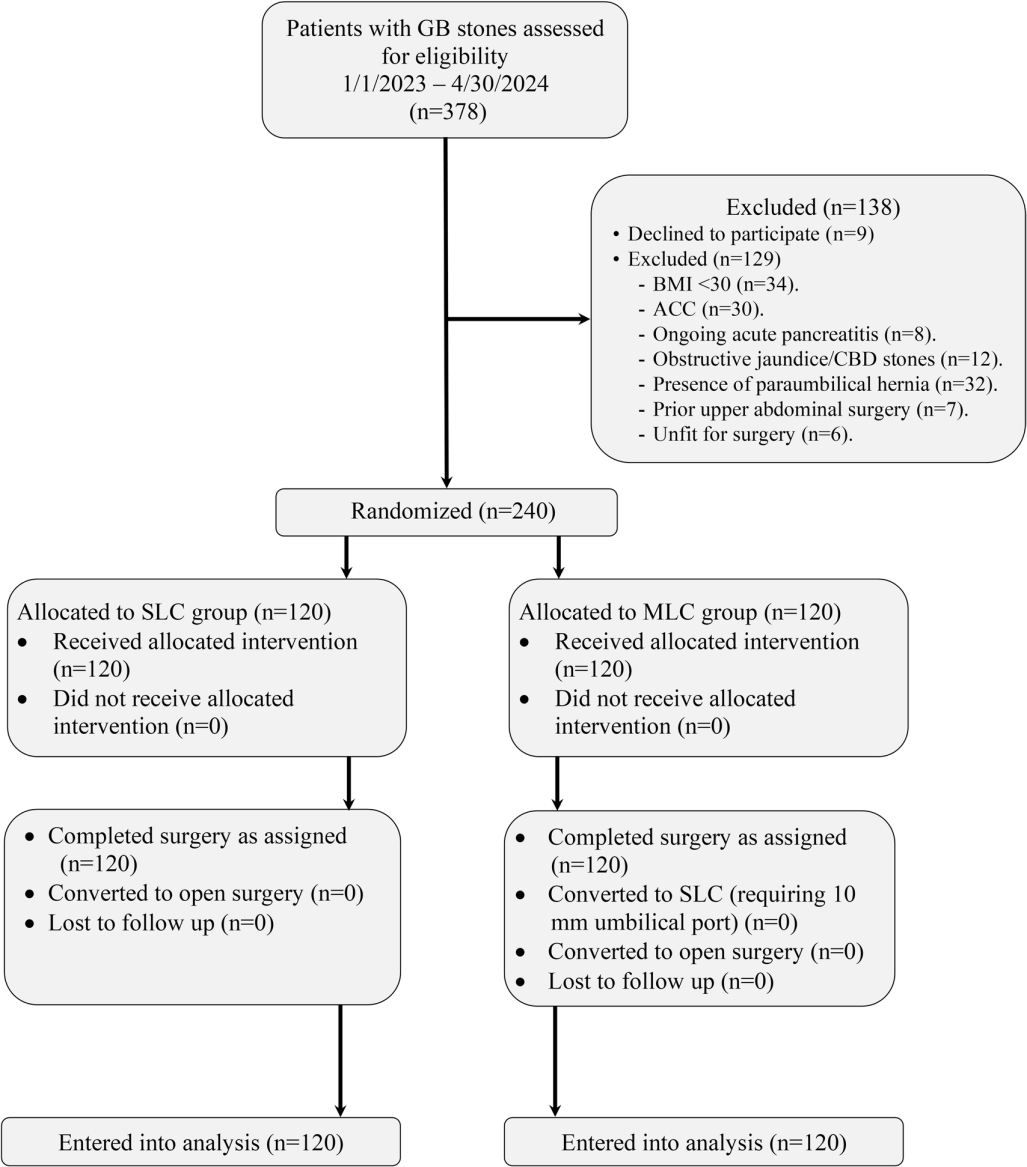
CONSORT chart of the study

Over 16-month period, 378 consecutive patients with symptomatic gallstone disease were screened for eligibility of inclusion in the study. Two hundred and forty patients met the inclusion and exclusion criteria and were randomly allocated to either of the 2 study groups with 120 patients per group as shown in Fig. 3.

Patient demography, preoperative laboratory, and imaging findings for the 2 groups are illustrated in Table 1. The groups were similar in terms of age, sex, BMI, and associated comorbidities.

**Table 1:** Patient demographic data, associated comorbidities, and preoperative investigations among SLC and MLC groups

Variables	SLC (*N* = 120)	MLC (*N* = 120)	*P*
Demographic data			
Age (y)	39.5 (28–48)	40 (31–50)	0.1
Sex, female	93 (77.5%)	87 (72.5%)	0.4
BMI (Kg/m^2^)	33 (31–35)	32 (31–34)	0.2
Comorbidities			
Diabetes mellitus	13 (10.8%)	20 (16.7%)	0.2
Hypertension	9 (7.5%)	16 (13.3%)	0.1
HCV infection	2 (1.7%)	1 (0.8%)	1
Prior history of			
Acute attack	14 (11.7%)	11 (9.2%)	0.5
Obstructive jaundice	2 (1.7%)	0	0.5
Pancreatitis	1 (0.8%)	0	1
Prior abdominal surgery	41 (34.2%)	55 (45.8%)	0.7
Appendectomy	14 (11.7%)	19 (15.8%)	0.3
Cesarean section	29 (24.2%)	37 (30.8%)	0.2
Others	3 (2.5%)	6 (5%)	0.5
Preoperative laboratory work-up			
Total bilirubin (mg/dL)	0.5 (0.4–0.7)	0.5 (0.3–0.7)	0.3
Direct bilirubin (mg/dL)	0.2 (0.15–0.21)	0.18 (0.13–0.21)	0.06
ALT (U/L)	22 (17–26)	23 (17–29)	0.3
AST (U/L)	20 (17–27)	22 (18–27)	0.5
ALP (U/L)	80 (66–99)	84 (70–98)	0.5
GGT (U/L)	26 (19–32)	24 (18–36)	0.9
Serum albumin	4 (3.8–4.3)	4 (3.8–4.2)	0.6
Hb (g/dL)	12.9 (12–13.9)	13 (11.9–14.1)	0.7
Total leukocyte count (× 10^3^/µL)	7.2 (6–8.6)	7.3 (5.8–8.9)	0.9
PMNL count (× 10^3^/µL)	3.8 (3.1–4.6)	4 (2.8–4.8)	0.9
Platelets (× 10^3^/µL)	269 (227–297)	258 (227–297)	0.6
INR	1 (1–1.1)	1 (1–1.1)	0.5
CRP (mg/L)	4.5 (2.5–8.4)	4.7 (2.7–7.5)	0.9
Ultrasound findings			
Number of gallstones			0.09
1	21 (17.5%)	35 (29.2%)	
2	15 (12.5%)	15 (12.5%)	
≥ 3	84 (70%)	70 (58.3%)	
Size of largest gallstone (mm)	8 (5–13)	9 (5–13)	0.8

Continuous variables reported as median (IQR)

Categorical variables reported as n (%)

Intraoperatively, all surgeries were successfully concluded via laparoscopy and no bail-out procedures were resorted to. None of the patients in the MLC group required conversion to conventional SLC and all surgeries were concluded using 5 mm umbilical port and 5 mm telescope. No biliary or visceral injuries were encountered. Gallbladder perforation occurred in a total of 9 patients (3.8%) and was comparable in the MLC and SLC groups (4.2% vs 3.3%, *P*=1). Stone spillage occurred in 3 patients (1.3%); 2 in the SLC group (1.7%) and 1 in the MLC group (0.8%), *P* = 1. Intraoperative bleeding from the GB liver bed was encountered in 2 patients in the MLC group (1.7%) compared to none in the SLC group (*P* = 0.5) which was successfully controlled by compression and bipolar electrocautery. Abdominal drain placement was similar among MLC and SLC groups (1.7% vs 2.5%, respectively, *P* = 1).

Postoperative complications according to Clavien–Dindo classification are illustrated in Table 2. Grade I was encountered in 6 patients (2.5%) who presented with one or more of the following: postoperative nausea and vomiting requiring antiemetic prescription (*n* = 3, 1.3%), postoperative fever (*n* = 5, 2.1%), epigastric port-site wound discharge requiring local drainage (*n* = 3, 1.3%), small epigastric port-site wound hematoma (*n* = 1, 0.4%), and umbilical port-site seroma requiring local aspiration/drainage (*n* = 3, 1.3%). Garde II was encountered in 2 patients (0.8%) in the form of epigastric port wound infection requiring repeated dressing and antibiotic coverage based on culture and sensitivity. Grade IIIA was encountered in 5 patients (2.1%) with deep-seated umbilical port-site wound infection requiring drainage, local debridement, and removal of remnant absorbable stitch under local anesthesia. Overall umbilical port-site wound complications were more encountered in SLC group as compared to MLC group (5.8% vs 0.8%, *P* = 0.07), however, the difference was not statistically significant.

**Table 2:** Study endpoints and postoperative outcomes among SLC and MLC groups

Variables	SLC (*N* = 120)	MLC (*N* = 120)	*P*
Total operative time (min)	52 (45–65)	46 (39–61)	0.005*
Abdominal access + GB dissection & retrieval	44 (35–55)	41 (35–56)	0.5
Port-wounds closure	9 (8–10)	5 (4–6)	< 0.001*
Pain score within first 24h postoperative			
2h	7 (5–8)	5 (4–6)	< 0.001*
6h	5 (4–6)	3 (2–4)	< 0.001*
12h	4 (3–5)	2 (1–2)	< 0.001*
24h (at time of discharge)	3 (2–3)	1 (1–2)	< 0.001*
Analgesic requirement during first 24h hospital stay			
2-dose ketorolac	52 (43.3%)	81 (67.5%)	< 0.001*
3-dose ketorolac	68 (56.7%)	39 (32.5%)	< 0.001*
Additional 1g paracetamol requirement	23 (19.2%)	10 (8.3%)	0.02*
Port-specific pain score on postoperative day 3			
Umbilical	2	0 (0–1)	< 0.001*
Epigastric	2 (1–3)	2 (2–3)	0.5
Right hypochondrial	0 (0–1)	0 (0–1)	0.8
Right lumbar	0 (0–1)	0 (0–1)	0.8
Duration of analgesic requirement at home (days)	5 (4–5)	3(2–3)	< 0.001*
Time required to return to normal daily activity (days)	5 (5–6)	3 (3–4)	< 0.001*
Umbilical port-site hernia	6 (5%)	0	0.03*
Clavien–Dindo classification of PO complications			
Grade I	4 (3.3%)	2 (1.7%)	0.7
Grade II	1 (0.8%)	1 (0.8%)	1
Grade IIIA	5 (4.2%)	0	0.06
Grade IIIB	6 (5%)	0	0.03*

^*^Statistical significance at *P* < 0.05

Continuous variables reported as median (IQR)

Categorical variables reported as *n* (%)

### Primary endpoint

Six out of 240 patients (2.5%) developed umbilical PSH after a mean of 12 months following surgery. They were exclusively encountered in the SLC group compared to none in the MLC group (5% vs 0, *P* = 0.03). Two patients presented with incarcerated umbilical PSH which required immediate intervention through laparoscopic intraperitoneal mesh repair. Recurrent periumbilical pain was the chief complaint among 3 patients, and the remaining patient complained of cosmetically disfiguring tiny bulge at the umbilical port-site wound. Interestingly, 5 out of these 6 patients with umbilical PSH suffered from umbilical port-site wound infection which was managed by combination of systemic antibiotic therapy, wound drainage, debridement, and frequent dressing.

### Secondary endpoints

While the median time required for abdominal access, GB dissection, and GB retrieval was similar in the MLC and SLC groups (41 min [IQR 35–56] vs 44 min [IQR 35–55], respectively, *P* = 0.5), the median time required for closure of the port-site wounds was significantly shorter in the MLC group (5 min, IQR 4–6) compared to SLC group (9 min, IQR 8–10), *P* < 0.001. Total operative time was significantly shorter in the MLC group (46 min, IQR 39–61) as compared to SLC group (52 min, IQR 45–65), *P* = 0.005.

**Fig. 4:**
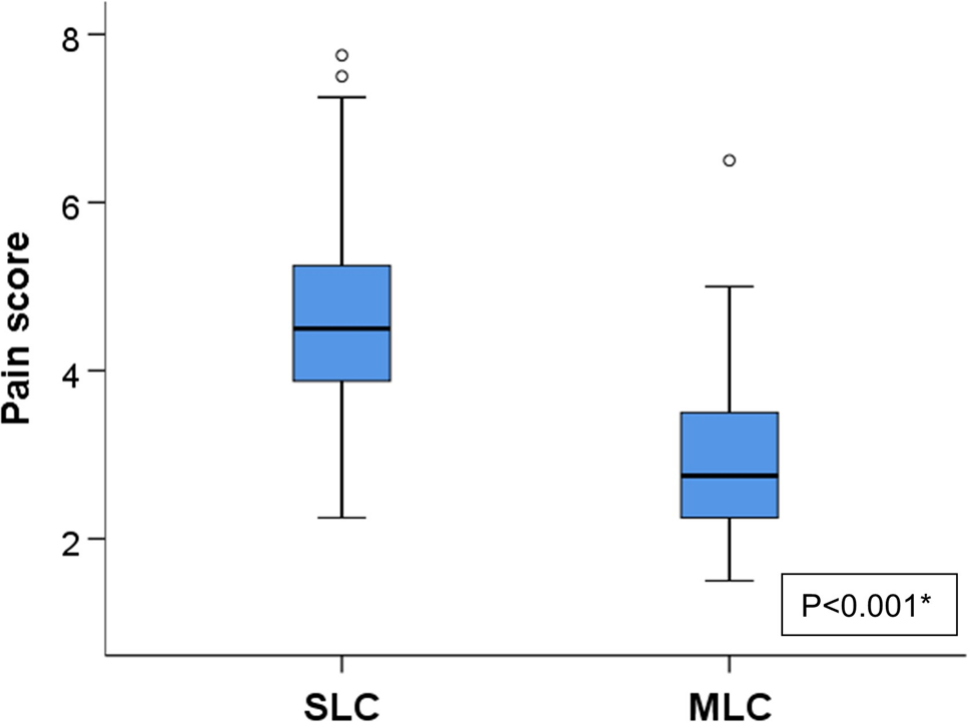
Average pain score during the first 24-h postoperative among both groups

Pain scores at 2-, 6-, 12-, and 24-h intervals were significantly lower among the MLC group compared to SLC group as illustrated in Table 2. The average pain score over the first 24 h post-surgery is illustrated in Fig. 4. Analgesic requirement was significantly lower among patients in the MLC group compared to those in the SLC group in terms of the number of ketorolac doses or additional paracetamol injections required during the first 24 h after surgery as shown in Table 2.

Port-specific pain scores assessed on the first follow-up visit were similar among both groups except for the umbilical port which had significantly lower score in MLC group as compared to SLC group (0 vs 2, *P* < 0.001). While tenderness over the upper abdominal port wounds (epigastric and two right subcostal) elicited during superficial palpation was similar among the 2 groups (83.3% vs 85.8%, *P* = 0.6), more patients expressed tenderness surrounding the umbilical port wound in the SLC group when compared to MLC group (71.7% vs 11.7%, *P* < 0.001).

The median duration of analgesic requirement at home was significantly shorter among MLC group compared to SLC group (5 vs 3 days, *P* < 0.001). Similarly, the median time required by patients to return to normal daily activities was shorter among the MLC group (5 vs 3 days, *P* < 0.001).

#### Patient and surgical team satisfaction

Patients in the MLC group had significantly higher satisfaction scores (9.5 ± 1) compared to SLC group (7.5 ± 1.8), *P* < 0.001 in terms of the size and cosmetic appearance of the umbilical wound when compared to the shape and size of the epigastric port (Supplemental Fig. 2). On the other hand, surgeon satisfaction with the use of 5 mm telescopes in MLC (9.1 ± 1.3) were inferior to those for using 10 mm telescopes in SLC group (9.4 ± 1.2), however, the difference was not statistically significant (*P* = 0.06). The reported notable differences between both telescopes were area of the visual field, image brightness, image sharpness, and accuracy of the fine anatomic details depicted (Supplemental Fig. 3).

## Discussion

This study evaluates the feasibility and outcomes of using 5 mm umbilical port and 5 mm telescope among obese patients undergoing LC while factoring in surgeon’s satisfaction to assess whether MLC could be performed on wider scale among obese patients.

It is not surprising that many would question the impact or outcome of reducing the size of only one port in LC. Nevertheless, in our study, using reduced 5 mm umbilical port was associated with significantly less postoperative pain during the first 24 h and lower umbilical-specific port wound pain after surgery. This evidently reflected on postoperative analgesic requirement which was significantly lower among patients in the MLC group. This could partly be explained by limited local tissue damage produced by the tiny 5 mm ports when compared to 10 mm or larger size ports. More importantly, the absence of facia closure for 5 mm ports could have predisposed to pain reduction owing to the absence of the usual tension exerted by the anchoring sutures on fascial tissues as in the case with routinely closed 10 mm ports. In their study comparing 5 mm vs 10 mm umbilical port in LC, Muneeb et al. reported significantly lower pain scores after 24 h and at discharge for the 5-mm-port group patients who consequently required significantly less ketorolac injection for pain control. [21] Similarly, Sarkar et al. in their study compared 5 mm and 10 mm umbilical port with 75 patients per group. They demonstrated significantly less postoperative pain at 24- and 72- hours post-surgery with the use of 5 mm umbilical port. [22]

Performing laparoscopic procedures in obese patients requires proper closure of the port-site wounds to avoid complications. It is established that port wounds ≥ 10 mm in size require fascia closure, however, this remains to be challenging and time consuming especially among obese patients attributed to the depth of fascia underlying a thick pad of subcutaneous fat of the abdominal wall. [23] Improper closure may predispose to PSH which became a recognized complication following laparoscopic procedures that could have serious consequences if associated with obstruction and/or strangulation. While no exact figure describes the actual incidence of PSH after laparoscopic surgery owing to the heterogeneity of procedures performed and devices used, the reported incidence of PSH following laparoscopic cholecystectomy ranges from 0.5 to 5.4% which are usually discovered after an average of 9 months post-surgery and up to 36 months from the date of surgery. [12, 13, 24, 25] Umbilical port is reported as the most common site for PSH especially when large ports ≥ 10 mm in diameter are used. Other recognized risk factors for developing PSH include obesity, port-site wound infection, improper port-closure technique, and the use of absorbable suture material. [12, 16, 24, 25] Increased intra-abdominal pressure, weak fascial integrity, and altered tissue dynamics precipitated by abdominal wall adiposity were considered to be the main factors behind the higher incidence of PSH among obese patients. [12, 15, 24, 26]

In our experience, umbilical PSH occurred in 2.5% of all the patients included in the study representing 5% of the patients within the SLC group. Although this remains within the incidence range reported in the literature, it lies closer to the upper limit. This could be explained by the presence of several risk factors contributing to PSH among such patient population. PSH was discovered in our experience after mean follow-up duration of 12 months post-surgery despite performing routine fascial closure of the 10 mm umbilical port for all patients in the SLC group. Fascial closure of ports ≥ 10 mm in diameter is recommended and became established to reduce development of PSH, however, it may not completely abolish its incidence. [12, 13, 24, 27, 28] In contrast, none of the patients in the MLC group developed umbilical PSH during the same follow-up period despite none of them requiring routine fascial closure of the umbilical port. The incidence of PSH in 5 mm ports remains to be rare with only few case reports that documented their occurrence. Hence, there is no clear guideline to date to recommend facial closure for such tiny ports except in pediatric patients. [24, 29–31]

Apart from postoperative pain and PSH, patients in the MLC group had shorter operative time (41 vs 44 min) which aligns with results from literature. Sarkar et al. in their study reported shorter operative time with the 5 mm port group compared to the 10 mm port group (47.3 vs 49.6 min). [22] In a gynecologic study carried out by Acton et al., they assessed the use of 5 mm vs 10 mm umbilical ports during total laparoscopic hysterectomy. [32] They similarly reported a significantly shorter operative time and less postoperative pain among patients in the 5 mm port group. This could be explained by the significant smaller size of the 5 mm ports which do not require fascial closure and require less time for skin closure when compared to 10 mm port wounds.

While postoperative hospital stay was not considered for comparison in this study since all the patients were successfully discharged as planned after 24 h from surgery, the duration of analgesic intake at home and the time required by patients to return to normal daily activities were significantly shorter among the MLC group as compared to SLC group. This could still be explained by the lower pain and less wound tension encountered at the 5 mm port-site wound, which could help the earlier return to normal daily activity observed in the MLC group. A couple of studies examined the effect of 5 mm umbilical port on postoperative hospital stay; however, none of them reported any significant difference when compared to those with conventional 10 mm umbilical ports. [22, 33]

In terms of cosmetic outcomes, several studies confirmed the superiority of MLC over conventional LC owing to the smaller port size. [8, 34] In our study, patients in the MLC group expressed significantly higher degree of cosmetic satisfaction with the umbilical wound compared to SLC group at 1, 3, and 6 months after surgery. Although the cosmetic results reported by Sarkar et al. at 6 months and 1 year where higher among the 5 mm umbilical port group, however, they did not reach statistical significance as compared to those with conventional 10 mm umbilical port. [22]

The compelling advantages of the small-sized 5 mm ports should, however, be considered in light of satisfaction of the surgical team who perform the surgery using the delicate 5 mm telescopes especially among obese patients. Four decades ago, the use of mini-telescopes was confined to diagnostic purposes and did not gain wide acceptance owing to their poor image quality, inferior resolution, and narrow visual field. [35] Nevertheless, the ongoing development in surgical equipment and fiberoptic technology resulted in considerable improvement in the quality of image transmitted by mini-telescopes. [36, 37] In this study, while our experience with the use of 5 mm telescopes was inferior to using 10 mm telescopes, overall surgeon satisfaction was considered comparable among both groups since it did not reach statistical significance. The 5 mm mini-telescopes were able to conclude LC surgeries in MLC group safely and efficiently without vascular or visceral injury while not impacting the flow of the procedure. In addition, we did not experience any form of difficulty that required replacement of the 5 mm telescopes with 10 mm ones for any of the patients in the MLC group and we were capable of dealing with challenging intraoperative events—as bleeding, properly and in timely manner.

However, several technical challenges appeared through our experience with the use of 5 mm telescopes that are worth mentioning. During the first few surgeries performed in MLC group, we were relatively annoyed by the significantly narrow visual field of the 5 mm telescopes and there was noticeable dimness of the image. Resolution/sharpness was also suboptimal with apparent granular haziness of the image rendering fine anatomic details poor and difficult to delineate. Such initital observations, however, did not persist with further practice as we got accustomed to the narrow visual field by time and our eyes became adapted to the quality of the image transmitted by such mini-telescopes.

At the conclusion of MLC procedure, evacuation of carbon dioxide gas from the abdominal cavity represented another challenge with 5 mm umbilical ports owing to their small size when compared to rapid exit of the gas with the 10 mm umbilical ports in SLC group. To overcome this, we had to use suction device tip placed through the 5 mm port or in between the fascial sutures of the already closed 10 mm epigastric port to help readily evacuate the gas under direct telescopic vision to prevent postoperative shoulder pain.

More importantly, the delicacy and slender nature of 5 mm telescopes renders them extremely fragile and hence great care is required while handling them among obese patients. This was obvious as they easily and readily bent while navigating them in every direction against resistance created by the heavy pendulous abdominal wall risking their damage especially when controlled by single hand of the assistant surgeon. Such critical technicality made it clear that two assistant surgeons would be required to assist the primary surgeon every time while performing MLC. One assistant was made responsible for grasping and manipulating the gallbladder fundus while allowing the second assistant (camera man) completely free-handed to navigate the camera/lens complex with both hands. The camera assistant surgeon had to use one hand to move the 5 mm umbilical port in the required direction initially and maintain it steady against the heavy abdominal wall while at the same time navigating the camera/lens complex in a passive manner to smoothly follow the actively moving port with the other hand. Such labor arrangement was, however, not required while performing SLC where just a single assistant surgeon was capable of handling the camera/telescope complex with right hand while retracting the GB with the left hand. This is attributed to the sturdier build and the bigger girth of the 10 mm telescopes which render them sustain the shear weight of the heavy abdominal wall while navigating them single handed with much lower risk of damage.

Consequently, it is quite evident that the durability of 5 mm telescopes is relatively inferior to 10 mm ones which could reflect on the cost of performing MLC related to the cost of frequent replacement of damaged 5 mm telescopes. However, such assumption may not prove accurate since throughout our experience over 16 months, there was not an instance where every MLC was complicated by damage of the 5 mm telescope that required replacement with new one. Nonetheless, the second assistant surgeon required each time while performing MLC may add an additional cost to the procedure. However, this could still be hampered by the shorter duration of surgery, less suture material requirement since no sheath closure is required, less postoperative analgesic requirement both during hospital admission and at home, and the earlier return to normal daily activities among MLC group patients. In addition, developing complications after surgery add extra cost to the index surgery should further postoperative care or intervention is required. The higher incidence of umbilical PSH among SLC group could be used as a surrogate to explain the potentially higher cost of SLC compared to MLC when management in terms of reoperation and mesh repair are considered.

In conclusion, our findings suggest that the use of 5 mm umbilical port and 5 mm telescope while performing LC is feasible and safe among obese patients provided that careful and proper patient selection is followed to avoid operating on patients with additional challenges which could further complicate the procedure. The advantages offered by using tiny ports and mini-telescopes in terms lower incidence of umbilical PSH, shorter operative time, less postoperative pain, lower analgesic requirement, earlier return to daily activities, and better umbilical wound cosmesis seem to outweigh their limitations which are usually overcome by repeated use and continued practice to build experience and adapt to the narrow field and inferior image quality. We suggest that expansion in the use of 5 mm umbilical ports and telescopes should be encouraged by surgeons while performing laparoscopic holecystectony for obese patients provided the availability of prior experience in minimally invasive surgery to allow for rapid adaptation and management of encountered intraoperative events.

This single center experience comprises a relatively small sample size with postoperative follow-up of 1 year which may represent a limitation. While comprehensive efforts were provided in this study beginning with performing surgeries, collection and accurate analysis of the data, and careful interpretation of results, we are aware that outcomes we reported require further validation with larger group of patients and more extended follow-up period > 1 year to avoid any chance of missing umbilical PSH with delayed presentation. The strength, however, of this study remains in the fact that all of the procedures were carried out by a single surgical team holding the same standard of care among all patients which guaranteed homogeneity and uniformity of the procedures performed and accuracy of the results reported across the study.

## Conclusion

Mini-laparoscopic cholecystectomy (MLC) for uncomplicated gallstone disease is safe and feasible in obese patients with the use of 5mm umbilical port and 5 mm telescope. It offers lower incidence of umbilical port complications including PSH, less postoperative pain, lower analgesic requirement, and better cosmesis.

## Supplementary Information

Below is the link to the electronic supplementary material.

Supplementary file1 (JPG 899 KB)

Supplementary file2 (JPG 1214 KB)

Supplementary file3 (JPG 1481 KB)
