# Supplementary figures and images for: 5 mm versus 10 mm umbilical port during laparoscopic cholecystectomy: do outcomes justify broader use in obese patients? A randomized controlled trial
Source: Surg Endosc. 2025 Aug 6;39(9):5927–38. doi: 10.1007/s00464-025-12016-5 (PMC12408780; doi:10.1007/s00464-025-12016-5)

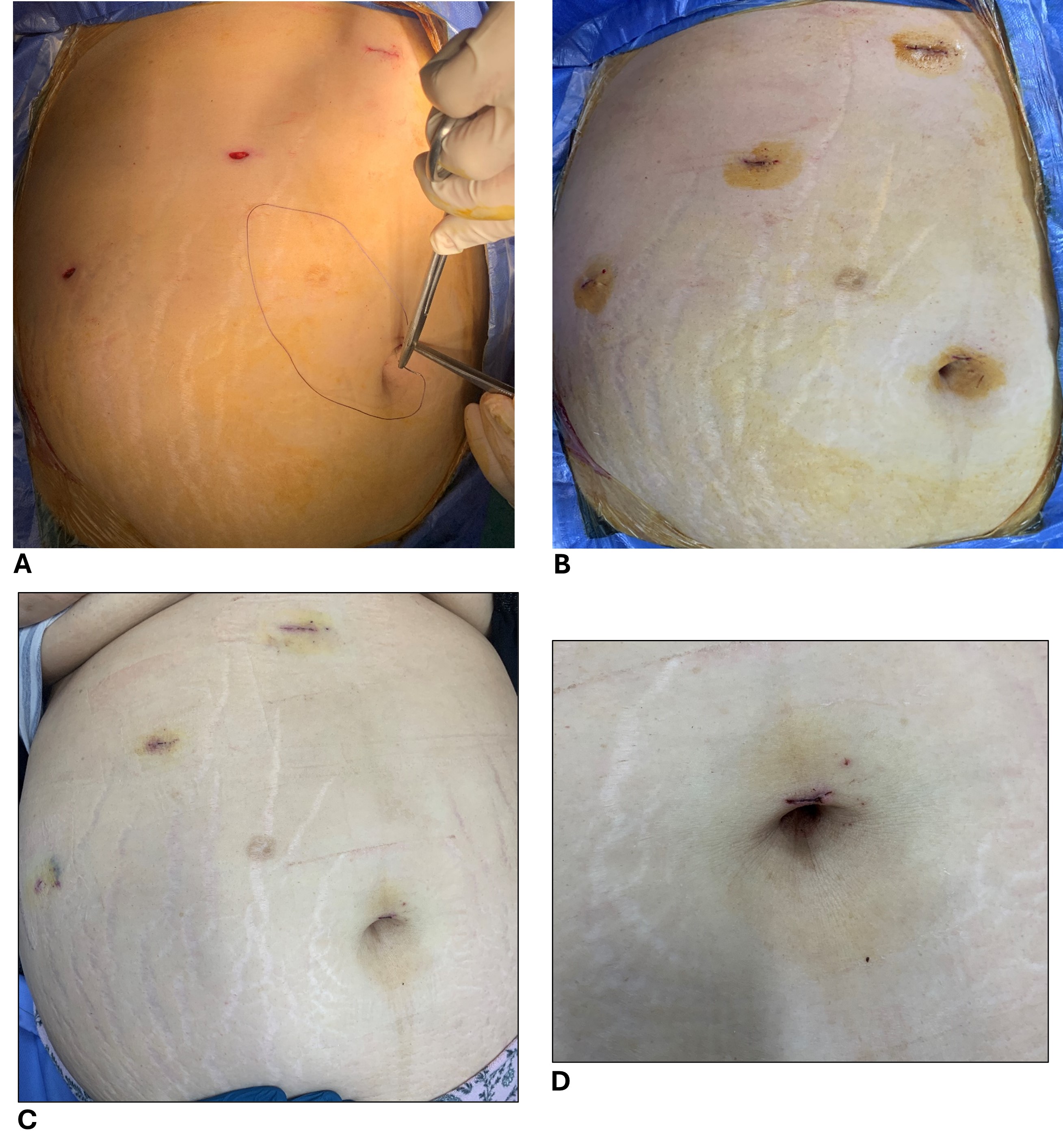

Supplement: Supplementary file 1 — Supplementary file1 (JPG 899 KB) [file 464_2025_12016_MOESM1_ESM.jpg]

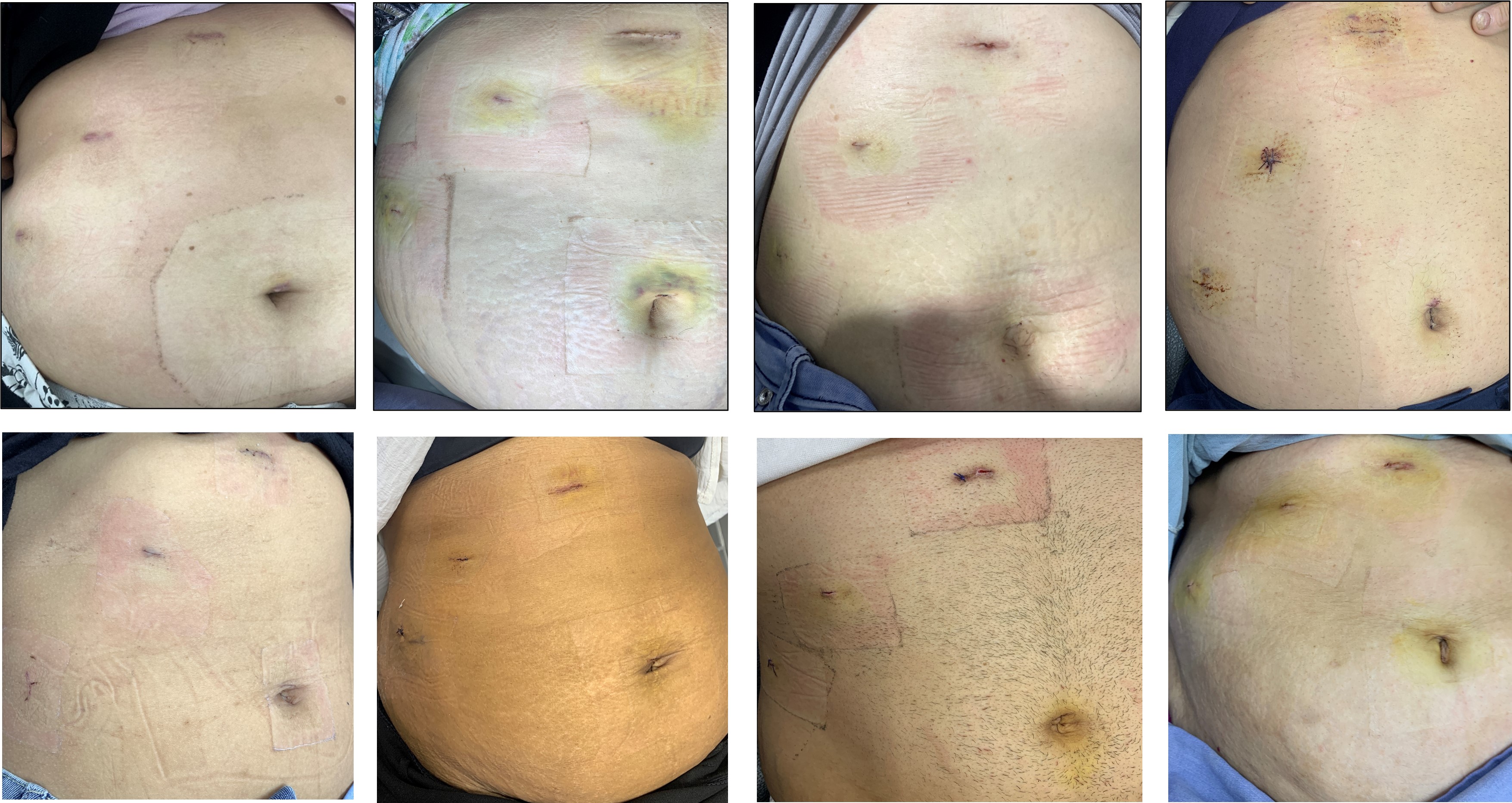

Supplement: Supplementary file 2 — Supplementary file2 (JPG 1214 KB) [file 464_2025_12016_MOESM2_ESM.jpg]

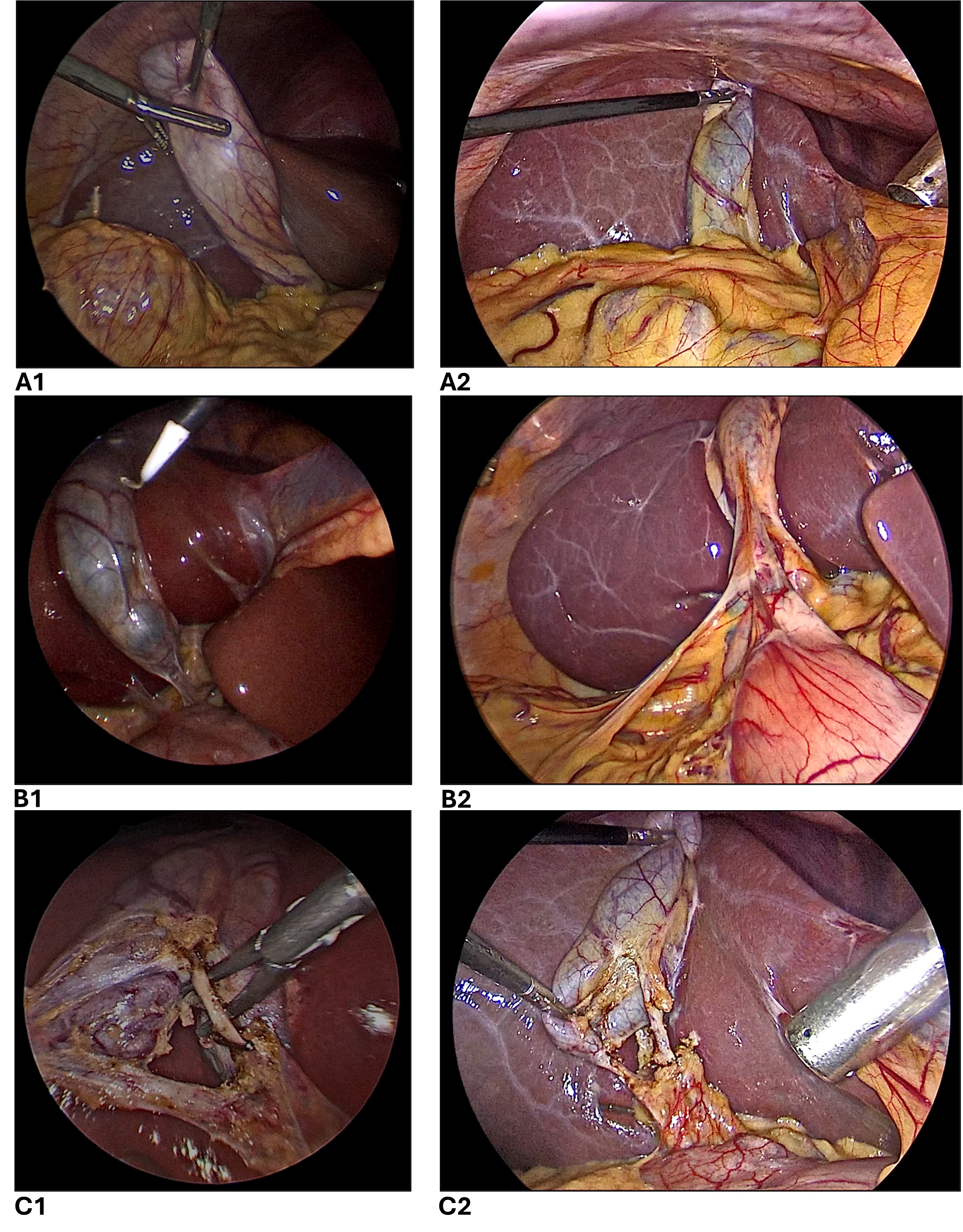

Supplement: Supplementary file 3 — Supplementary file3 (JPG 1481 KB) [file 464_2025_12016_MOESM3_ESM.jpg]
